# Effects of the Presence of Pseudoexfoliation on Intraocular Pressure and Retinal Nerve Fiber Layer Thickness in Patients with Macular Degeneration Receiving Intravitreal Ranibizumab †

**DOI:** 10.3390/clinpract12010009

**Published:** 2022-01-19

**Authors:** Hatice Daldal, Melike Balikoglu Yilmaz

**Affiliations:** 1 Department of Ophthalmology, Faculty of Medicine, Usak University, Usak 64000, Turkey; 2 Department of Ophthalmology, Faculty of Medicine, İzmir Katip Celebi University, İzmir 35620, Turkey; drmelikebalkoglu@yahoo.com

**Keywords:** intraocular pressure, intravitreal treatment, macular degeneration, pseudoexfoliation, ranibizumab, retinal nerve fiber layer

## Abstract

Aims: In the present study, we aimed to compare the effect of intravitreal ranibizumab (IVR) treatment on intraocular pressure (IOP) and retinal nerve fiber layer (RNFL) thickness in patients with age-related macular degeneration (AMD) with and without pseudoexfoliation (PEX). Materials and Methods: A total of 24 patients, 12 with PEX (12 eyes) and 12 without PEX (12 eyes), receiving IVR treatment for neovascular AMD between June 2017 and June 2019, were included in the study. Exclusion criteria were composed of the history of glaucoma, uveitis, intravitreal steroid administration, pars plana vitrectomy surgery, and less than three IVR injections. Such criteria as age, gender, follow-up times, number of injections administered, IOP, and RNFL thickness before the first injection and one month after the last injection were also recorded. Results: Age, gender, follow-up time, and the number of injections were similar in groups with and without PEX (*p* > 0.05). While mean post-treatment IOP values were not significantly higher in the PEX group (14.50 ± 3.06 vs. 12.91 ± 1.83 mmHg, *p* = 0.065), the values were significant for the non-PEX group (13.25 ± 2.76 vs. 11.83 ± 2.69 mmHg, *p* = 0.01), and these values were within normal IOP limits. Additionally, RNFL thickness was significantly thinner after treatment in both groups (91.41 ± 7.14 vs. 94.00 ± 6.76 in those with PEX; 95.58 ± 5.91 vs. 97.66 ± 6.89 in those without PEX; *p* < 0.05). The decrease in RNFL thickness in the PEX group was 2.58 ± 1.62 µ and in the non-PEX group was 2.08 ± 1.98 µ. However, there was no statistically significant difference between the two groups in terms of RNFL thinning (*p* = 0.505). Discussion: Ranibizumab may reduce RNFL thickness in patients with PEX. Longer-term studies including larger populations are necessary for understanding IOP and RNFL changes after anti-vascular endothelial growth factor (anti-VEGF) injection.

## 1. Introduction

Pseudoexfoliation (PEX) syndrome is a systemic disease characterized by the accumulation of fibrillar material in the anterior lens capsule, ciliary epithelium, zonules, iris, and corneal endothelium. The pathogenesis of PEX is controversial, and various mechanisms are held responsible. First, the elastic microfibril hypothesis and the overproduction of elastic fibrils can lead to the accumulation of PEX material. Second, pseudoexfoliative material may consist of the fusion of metabolites accumulated as a result of abnormal metabolism of basal membrane proteoglycans and iris glycosaminoglycans. Finally, transforming growth factor β1 overexpression and imbalance between matrix metalloproteinases and tissue inhibitory matrix metalloproteinases may be responsible [1].

Lysyl-oxidase-like 1 (LOXL1) gene mutations have been determined as a risk factor for PEX. Additionally, caffeine intake and vitamin deficiency may also play a role in the pathogenesis of PEX [2]. PEX has been identified in intraocular, periocular, non-ocular tissues. Such findings support that PEX is a systemic condition [3,4,5].

Choroidal neovascularization is the leading cause of serious vision loss in age-related macular degeneration (AMD) [6]. Vascular endothelial growth factor (VEGF) is considered to be a factor playing the most important role in choroidal neovascularization [7]. Ranibizumab inhibits all isoforms of VEGF-A and VEGF165, VEGF121, and VEGF110 [8]. Ranibizumab is efficient in reducing cell proliferation, new blood vessel formation, and vascular leakage [9]. Ranibizumab has been approved by the Food and Drug Administration (FDA) in neovascular AMD since 2006 [10].

Here, we aimed to evaluate the effect of the presence of pseudoexfoliation on intraocular pressure (IOP) and retinal nerve fiber layer (RNFL) thickness in patients with neovascular age-related macular degeneration (AMD) administered intravitreal ranibizumab injections (IVR).

## 2. Materials and Methods

The study was designed as a retrospective study including a total of 24 patients, 12 with PEX (12 eyes) and 12 without PEX (12 eyes), treated with IVR due to wet (neovascular) type AMD between June 2017 and June 2019 were included in the study. The study approved the tenets of the Declaration of Helsinki. Ethics committee approval was obtained before the study (9.06.2021/12) and written informed consent was obtained from all of the patients. Exclusion criteria consisted of the history of glaucoma, uveitis, intravitreal steroid administration, YAG laser capsulotomy within three months, pars plana vitrectomy surgery, photodynamic therapy, and less than three IVR injections, such systemic diseases as diabetes mellitus and hypertension. The patients were divided into two groups as age-related macular degeneration with and without PEX. Additionally, such criteria as age, gender, follow-up time, number of injections administered, IOP, and RNFL thickness of patients were also recorded before the first injection and one month after the last injection.

All patients underwent a comprehensive ophthalmologic examination. While the best-corrected visual acuity (BCVA) was evaluated with the Snellen chart, IOP was measured with the Goldmann applanation tonometry, and the biomicroscopic anterior segment examinations and fundus examinations were performed using a 90D lens. The measurements of the central macular thickness (CMT) and RNFL thickness were performed with the spectral-domain optical coherence tomography OCT (Cirrus HD-OCT 4000; Carl Zeiss Meditec, Jena, Germany), and the fundus fluorescein angiography (FFA) was also performed for each patient.

Each patient in the study received a loading dose of 0.05 mL intravitreal ranibizumab (Lucentis; Genentech USA Inc., San Francisco, CA, USA/Novartis Ophthalmics, Basel, Switzerland). Thereafter, the treatment was continued based on the activity of choroid neovascularization (decreased visual acuity, fluid in OCT, presence of retinal hemorrhage) under the treatment protocol. The injection procedure was carried out by the same physician (HD) in the operating room under sterile conditions. The statistical analyses were evaluated using The Statistical Package for Social Sciences for Windows 22.0 program (SPSS Inc., Chicago, IL, USA), as well as the chi-square and Willcoxon tests. In the study, a *p*-value of less than 0.05 was considered to be significant.

## 3. Results

The mean age of the patients was 69.91 ± 7.10 years in the PEX group and 71.33 ± 7.47 years in the non-PEX group. In Table 1, it can be seen that the two groups are equal in terms of distribution of age values (*p* = 0.524). There were six female and six male patients in PEX and non-PEX groups, and there was no significant difference in both groups. The follow-up time was 15.91 ± 4.12 months (min–max: 12–24) in the PEX group and 16.33 ± 4.14 months (min–max: 12–24) in those without PEX. In Table 1, the populations in both groups are seen to be equal in terms of distribution of follow-up times (*p* = 0.814). The number of injections was 7.00 ± 2.33 in the PEX group and 7.83 ± 2.32 in the non-PEX group. As seen in Table 1, the two groups are equal in terms of the distribution of the number of injections (*p* = 0.382). Therefore, it can be concluded that PEX and non-PEX groups had similar values in terms of age (*p* = 0.524), gender (*p* = 1.0), follow-up time (*p* = 0.814), and the number of injections (*p* = 0.382) (Table 1).

While the mean post-treatment IOP value was not significantly higher in the PEX group (14.50 ± 3.06 vs. 12.91 ± 1.83 mmHg, *p* = 0.065), the value was found as significant for the non-PEX group (13.25 ± 2.76 vs. 11.83 ± 2.69 mmHg, *p* = 0.01), and these values were within normal IOP limits (Table 2). Furthermore, RNFL thickness was significantly thinner after the treatment in both PEX and non-PEX groups (91.41 ± 7.14 vs. 94.00 ± 6.76 in the PEX group; 95.58 ± 5.91 vs. 97.66 ± 6.89 in the non-PEX group, *p* < 0.05) (Table 2).

The decrease in RNFL thickness in the PEX group was 2.58 ± 1.62 µ and in the non-PEX group was 2.08 ± 1.98 µ. However, there was no statistically significant difference between the two groups in terms of RNFL thinning (*p* = 0.505).

## 4. Discussion

PEX syndrome is associated with a lower prevalence of neovascular age-related macular degeneration [11]. In our 2-year study, there were 12 AMD patients with PEX. In the study performed by Kling et al., a significant relationship was reported between the statistical increase of PEX and AMD with age [12].

In the study by Allingham et al., PEX was stated to be strongly associated with glaucoma but not with either AMD or systemic disease [13]. Similarly, it was found that PEX syndrome and exfoliation glaucoma were not associated with a higher frequency of AMD when controlling for age in the study of Tarkkanen et al. [14].

Short-term transient increases were shown in IOP, returning to the baseline 30–60 min after the intravitreal injections. Such increases in IOP are considered to be caused by the mechanical effect of injecting a volume of fluid into the relatively constrained space of the globe [15,16,17,18]. In ANCHOR, MARINA, VIEW1, VIEW2 studies, a sustained IOP increase was not observed after anti-VEGF injection [15,19,20]. In other studies, however, a sustained high IOP was shown to be related to anti-VEGF injections.

Bakri et al. reported for the first time as case series consisting of four patients with delayed and sustained high IOP after ranibizumab injection [21]. The prevalence of long-term increased IOP in those receiving an intravitreal anti-VEGF injection is between 2–11%, 4.7% overall [22].

Although the exact mechanism of sustained high IOP due to anti-VEGF injection remains unknown, multifactorial processes and different factors have been implicated as the causes of the condition. Two causes were considered to be the culprits: firstly, the mechanical blockages of the trabecular meshwork and Schlemm’s canal with anti-VEGF agents have been found responsible for the recurrence of transient high IOP after injection [23,24,25], and secondly, the drug-related complications, and the inflammation such as trabeculitis and uveitis, and its cytotoxic effects on the trabecular meshwork in high concentrations have been demonstrated to be responsible in vitro studies [21,26]. The inhibition of nitric oxide production by anti-VEGF drugs is considered to play a role in the sustained high IOP [27]. In another study where Ucgul et al. compared the effects of intravitreal bevacizumab and IVR on IOP in patients with and without PEX, the definition of sustained elevation of IOP was demonstrated to be varied. In the study, the sustained elevation of IOP had been accepted as the elevated IOP above 21 or 5 mmHg from baseline for at least two visits. Ucgul et al. found that bevacizumab treatment may cause more increase in IOP among the patients with PEX syndrome, and ranibizumab seemed to be safer than bevacizumab in terms of IOP control [28].

As different from previous studies, we also evaluated the effect of ranibizumab treatment on RNFL thickness in addition to IOP in our study. On the other hand, the number of the participants was small as 12 patients with the coexistence of PEX and AMD in our study performed within two years. Although increasing after the injections, IOP returned to normal limits, and no sustained high IOP was found in any of our patients.

In the study of Bakri et al. where the ranibizumab treatment was compared between the patient and the control groups, the prevalence of sustained high IOP was found as 39.9, 10.9, and 26.1% in the ranibizumab group and 29.1, 5.1, and 13.6% among the controls, respectively, with IOP ≥ 21 mmHg, IOP ≥ 25 mmHg, IOP ≥ 21 mmHg, and ≥6 mmHg above the baseline values [29].

In the study of Choi et al., sustained or unsustained high IOP was reported not to be rare after intravitreal anti-VEGF injections. In the study, the researchers evaluated 155 eyes of 127 patients and detected that IOP increased in 14 eyes (9.4%); in seven of those eyes, 5.5% developed a sustained high IOP (above 25 mmHg on two visits requiring glaucoma treatment). However, eight of these patients required topical drop, and only one patient required glaucoma surgery. It was also stated that there was no association with the frequency and number of injections or drugs used for those with high IOP [30].

In the present study, the mean post-treatment IOP value was observed not to be significantly higher in the PEX group; however, the mean post-treatment IOP value was significant for the non-PEX group, and those values were within normal IOP limits. Although increasing after the injection, IOP was still within the normal limits, and no sustained elevation was found in any of the patients with IOP. In addition, RNFL thickness was significantly thinner after the treatment in both PEX and non-PEX groups. However, there was no statistically significant difference between the two groups. Although the reason for the decrease in RNFL thickness is unclear, the increase in IOP after the treatment is likely to be an effect. At the same time glaucoma is significantly associated with neovascular AMD [31]. AMD and glaucoma can have a synergistic effect in determining the low vision, and glaucoma is often underreported in these patients.

## 5. Conclusions

In conclusion, RNFL thickness was significantly thinner after IVR injection in AMD patients with PEX and without PEX. Ranibizumab may reduce RNFL thickness in those with PEX.

The main limitation of our study is the low sample size. We consider that longer-term studies including larger populations are necessary to understand the changes developing in IOP and RNFL after the anti-VEGF injection. Comprehensive studies are likely to reveal the molecular interaction between ranibizumab and PEX material or other mechanisms hypothesized in other studies.

## Figures and Tables

**Table 1:** Demographic and clinical characteristics of groups.

	PEX	Non-PEX	*p*
Age (years) (mean ± SD)	69.91 ± 7.10	71.33 ± 7.47	0.524
Gender (n) F/M	6/6	6/6	1.0
Follow-up time (mean ± SD)	15.91 ± 4.12	16.33 ± 4.14	0.814
Number of injections (mean ± SD)	7 ± 2.33	7.83 ± 2.32	0.382

F: Female, M: Male, PEX: Pseudoexfoliation, SD: Standard deviation.

**Table 2:** Comparisons of pre- and post-treatment IOP and RNFL thickness.

	Pre-Treatment	Post-Treatment	*p*
IOP (mmHg) in PEX Group	12.91 ± 1.83	14.50 ± 3.06	0.065
IOP (mmHg) in Non-PEX Group	11.83 ± 2.69	13.25 ± 2.76	0.01 *
RNFL thickness (µ) in PEX Group	94.00 ± 6.76	91.41 ± 7.14	0.004 *
RNFL thickness (µ) in Non-PEX Group	97.66 ± 6.89	95.58 ± 5.91	0.007 *

* Significance at the 0.05 level, IOP: Intraocular pressure, PEX: Pseudoexfoliation, RNFL: Retinal nerve fiber layer.
